# Selected *E2F2* Polymorphisms in Oral and Oropharyngeal Squamous Cell Carcinoma

**DOI:** 10.1155/2021/8098130

**Published:** 2021-03-30

**Authors:** Karolina Gołąbek, Krzysztof Biernacki, Jadwiga Gaździcka, Joanna K. Strzelczyk, Katarzyna Miśkiewicz-Orczyk, Łukasz Krakowczyk, Natalia Zięba, Paweł Kiczmer, Zofia Ostrowska, Maciej Misiołek

**Affiliations:** ^1^Department of Medical and Molecular Biology, Faculty of Medical Sciences in Zabrze, Medical University of Silesia in Katowice, Zabrze 41-808, Poland; ^2^Department of Otorhinolaryngology and Oncological Laryngology, Faculty of Medical Sciences in Zabrze, Medical University of Silesia in Katowice, Zabrze 41-800, Poland; ^3^Clinic of Oncological and Reconstructive Surgery, Maria Sklodowska-Curie National Research Institute of Oncology, Gliwice 44-102, Poland; ^4^Department of Pathomorphology, Faculty of Medical Sciences in Zabrze, Medical University of Silesia in Katowice, Zabrze 41-800, Poland

## Abstract

Oral squamous cell carcinoma (OSCC) and oropharyngeal squamous cell carcinoma (OPSCC) are subgroups of head and neck squamous cell carcinoma. E2F Transcription Factor 2 (E2F2) could contribute to cancer development, because it plays a critical role in many cellular processes, including the cell cycle, proliferation, differentiation, DNA damage response, and cell death. In the current study, we assessed the associations of five E2F2 polymorphisms (rs6667575, rs3218121, rs3218211, rs3218148, and rs3218203) with OSCC and OPSCC and influence on the TNM staging and grading. This is the first such survey to concern the European population. The study included 94 primary tumour samples following surgical resection from patients, whereas the control group consisted of 99 healthy individuals. We tried a matching of cases and controls for age and sample size. DNA samples were genotyped by employing the 5′ nuclease assay for allelic discrimination. Our results suggested that the most significant difference between the control group and the cancer group was the A/G heterozygote for rs3218121. Samples containing this genotype were mostly found in the control group. In our samples, rs6667575, rs3218121, rs3218211, and rs3218148 polymorphisms may affect the course of OSCC and OPSCC, while rs3218203 was not associated with OSCC and OPSCC. However, further studies are warranted to confirm our findings.

## 1. Introduction

Head and neck squamous cell carcinoma (HNSCC) is an epithelial tumour with more than 800 000 cases diagnosed each year [1, 2] with the overall 5-year survival rate of approximately 40-50% [2]. Oral squamous cell carcinoma (OSCC) is the most common type of HNSCC. HNSCC is also common in the oropharynx (OPSCC) [3]. The incidence of these two types of HNSCC is still increasing [4, 5]. Exposure to tobacco and moderate alcohol consumption are important etiological factors in HNSCC carcinogenesis. Infections with high-risk human papillomaviruses (HPV) are responsible for an increasing proportion of OSCC [6, 7]. Other factors include poor oral hygiene, exposure to carcinogenic chemicals, and poor diet [6, 8, 9]. Another potential group of risk factors is related to endogenous factors such as genetic predisposition [9]. Single nucleotide polymorphisms (SNPs) are typical examples of this group [10]. Some studies showed that E2F Transcription Factor 2 (E2F2) promoter polymorphisms, which affect the expression of *E2F2*, are significantly associated with increased risk of many cancers [11–15]. E2F Transcription Factor 2 (E2F2) could contribute to cancer development, because it plays a critical role in many cellular processes, including the cell cycle, proliferation, differentiation, DNA damage response, and cell death [16–18]. The transcription factors of the E2F family are the dimers of E2F and DP proteins. It is known that E2F2 is a member of this transcription factor family and these factors have already been identified to bind to the RB protein. The RB/E2F pathway plays an important role as a regulator of cell proliferation at the G1/S checkpoint. E2F-DP complexes may promote cell entry into the S phase. As long as the E2F-DP complex is inactivated, the cell is stopped in phase G1. When RB binds to E2F, the resulting complex acts as a growth promoter [19].

One study was found which presented the relationship between five *E2F2* polymorphisms (rs6667575, rs3218121, rs3218211, rs3218148, and rs3218203) and HNSCC (of different locations) in the population from USA (white, not Latino) [20]. In Europe, we have no data regarding those issues. Because of the expected differences in genotype frequencies between ethnic groups in the current study, we assessed the associations of *E2F2* polymorphisms rs6667575, rs3218121, rs3218211, rs3218148, and rs3218203 with the risk of OSCC and OPSCC and influence on the TNM staging and grading in the Polish population.

## 2. Materials and Methods

### 2.1. Patients and Samples

The study included 94 primary tumour samples obtained from Polish patients following surgical resection at the Department of Otorhinolaryngology and Oncological Laryngology, Faculty of Medical Sciences in Zabrze, Medical University of Silesia in Katowice, and the Maria Sklodowska-Curie National Research Institute of Oncology (formerly known as the Maria Sklodowska-Curie Memorial Cancer Centre and Institute of Oncology), Gliwice, Poland. All tumours collected during surgery were OSCC and OPSCC (following the ICD-10 classification—C01: 42; C04.8-C05.2: 23; C09: 14; C05.8-C06.2: 13; C00.5: 1; and C03.0-C03.1: 1). Tumour staging was based on the American Joint Committee on Cancer (AJCC, version 2007) [21, 22] and WHO Classification of Head and Neck Tumours [23]. All samples were immediately frozen at -80°C until DNA extraction. These tumour samples were histologically confirmed by pathologists and were classified as primary tumours. Patients included in this study had no history of preoperative radio- or chemotherapy. The control group consisted of 99 healthy individuals without a history of cancer at any site or oral precancerous disease. We tried a matching of cases and controls for age and sample size. All patients and controls were Caucasians who lived in Poland. The study was approved by the Bioethics Committee of the Medical University of Silesia (Katowice, Poland; approval no. KNW/022/KB1/49/16 and no. KNW/002/KB1/49/II/16/17) and the Institutional Review Board on Medical Ethics of the Maria Sklodowska-Curie Memorial Cancer Centre and Institute of Oncology in Gliwice (Poland; approval no. KB/493-15/08 and no. KB/430-47/13).

### 2.2. DNA Extraction and SNP Analyses

The methodology of DNA isolation and SNP analyses was also described for the first time in the previous study [24]. Genomic DNA was extracted from each tumour sample (20 mg) by DNeasy Blood & Tissue Kits (Qiagen, Hilden, Germany) according to the manufacturer's instructions, after tissue homogenization in a FastPrep®-24 instrument using Lysing Matrix A tubes (MP Biomedicals, Solon, CA, USA). In the control group, the DNA was extracted from swabs taken from oral mucous membranes using a Swab-Extract DNA Purification Kit (EURx, Gdansk, Poland), according to the manufacturer's instructions. Next, high-quality DNA was eluted in a low-salt buffer containing 10 mM Tris-HCl, pH 8.5. The qualitative and quantitative analysis of all isolated DNA was performed by spectrophotometry in a Biochrom WPA Biowave DNA UV/Vis Spectrophotometer (Biochrom, Cambridge, UK), according to the manufacturer's instructions.

Genotyping was conducted with a QuantStudio 5 Real-Time PCR System (Applied Biosystems, Foster City, CA, USA). The reaction solution contained 5 *μ*g DNA (5.5 *μ*l), 12.5 *μ*l TaqMan Genotyping Master Mix (Applied Biosystems, Foster City, CA, USA), and 1.25 *μ*l TaqMan Genotyping Assay (Applied Biosystems, Foster City, CA, USA). SNP calling was read out automatically in QuantStudio Design and Analysis Software v1.5.1 (Applied Biosystems, Foster City, CA, USA). The types of polymorphisms and the primers used in the study are listed in Table 1.

### 2.3. Statistical Analysis

The Hardy-Weinberg equilibrium was used for the entire study group and separately for the control and cancer groups. The Kruskal-Wallis test was used for comparison for gender, age, smoking, and alcohol consumption between the control and patient groups. The Fisher exact test was used to search for selected parameters (gender, smoking, and alcohol consumption) with a significant influence on the cancer odds and genotypes with a significant influence on the cancer risk and TNM staging and grading in the cancer cohort. All statistical analyses were performed using Power Analysis Software STATISTICA v. 13.36.0 (StatSoft, Krakow, Poland); *α* = 0.05 was used in all tests.

## 3. Results

### 3.1. Patient Characteristics

A total of 94 patients with OSCC and OPSCC were included in this study. Their clinical parameters are given in Table 2. The average age was 62 years (range: 15–78 years). There were 68 (73%) men and 26 (27%) women; 65 (68%) patients were smokers; 64 (67%) reported alcohol consumption; 51 (54%) were both smokers and alcohol users. The control group consisted of 99 healthy individuals. The average age of the controls was 52.92 years (range: 18–69 years). This group comprised 22 (22%) men and 77 (78%) women, of whom 20 were smokers (20%), 67 were drinkers (68%), and 20 were both tobacco and alcohol users (20%).

### 3.2. SNP Distribution of E2F2 Polymorphism

The SNP distribution is presented in Table 3. All SNP distributions followed the Hardy-Weinberg equilibrium with the exception of rs3218121. Patients with rs3218121 had significantly fewer heterozygotes than the control group, while the control group had no reference homozygotes, which could be due to the limited number of samples.

### 3.3. Demographics and Risk Factors for Study Subjects

The most significant factors in our study that could contribute to cancer were gender (females had significantly lower odds of cancer than males, OR = 9.15, *p* < 0.001) and smoking (smoking increased the odds of cancer, OR = 0.11, *p* < 0.001). However, in the case of all parameters (gender, smoking, and alcohol consumption), there was a significant difference between the control and cancer groups (Kruskal-Wallis test, *p* < 0.001 for all tested parameters).

### 3.4. Effects of E2F2 Polymorphism on Odds OSCC and OPSCC

Table 4 shows the associations of each individual polymorphism with odds of OSCC and OPSCC. The most significant differences between the control group and the cancer group were found in the case of the A/G heterozygote for rs3218121. Samples containing this genotype were mostly found in the control group. Therefore, this genotype seemed to be significant in cancer odds reduction (OR = 127.51, *p* < 0.001).

### 3.5. Associations of E2F2 Genotypes with TNM Staging and Grading in the OSCC and OPSCC and Cohort Groups

We used the Fisher exact test separately in the control and cancer groups, as well as in the combined cohort with the assumption that TNM staging and grading were absent in controls.

**Figure 1:**
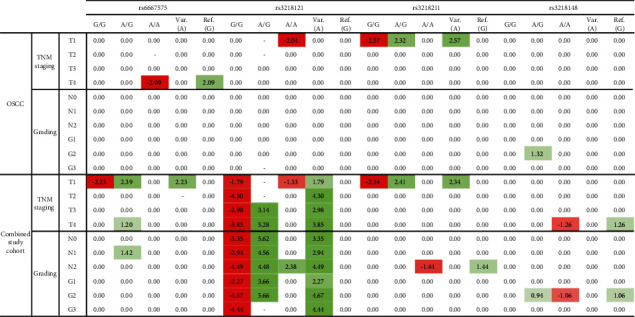
Heatmap represents the influence of *E2F2* genotypes on the TNM staging and grading in the OSCC and OPSCC and cohort groups.

The heat map represents the influence of a specific genotype based on the odds of specific TNM staging and grading. Significant values (*p* ≤ 0.05) are presented as log_2_ (OR), insignificant values (*p* > 0.05) as 0 (no change in odds), and incalculable cases as ‘-'. Cases that significantly increased the odds of specific TNM staging and grading are marked in red, whereas a significant decrease in the odds is marked in green. Values in each case represent log_2_ of the Fisher exact odds ratio. Therefore, negative values represented how much higher the odds of specific TNM staging and grading were for a specific genotype or allele, while positive values represented how much lower the odds of specific TNM staging and grading were for a specific genotype or allele. The heat map is presented in Figure 1.

In this study, rs3218211 is the only polymorphism that showed a consistent increase in the odds of T = 1 for the G/G homozygote in both the cancer and cohort groups. There was also a consistent decrease in odds of T = 1 for both the A/G heterozygote and A allele. However, in the case of the A/A homozygote, we observed an increase in the odds of N = 2 in the whole study cohort, with a corresponding decrease in the odds for the G allele. The impact of A/G for rs3218148 in cancer patients was limited to a decrease in the odds of G = 2, which could also be found in the combined cohort. However, in the whole study cohort, an additional increase in the odds of G = 2 could be observed for the A/A homozygote, and the presence of the G allele decreased those odds. A similar observation was seen when T = 4 was analyzed. A/G rs6667575 showed a consistent increase in the odds of T = 1 for the G/G homozygote in the whole cohort groups. In the case of cancer patients, only the odds of T = 1 were significantly higher for the A/A rs3218121, which could be additionally observed in the whole study cohort.

## 4. Discussion

It is known that E2F2 is a member of the E2F transcription factor family and these factors have already been identified to bind to the RB protein. The RB/E2F pathway plays an important role as a regulator of cell proliferation at the G1/S checkpoint [19]. Therefore, it seems warranted to determine the significance of *E2F2* polymorphisms in carcinogenesis. To the best of the authors' knowledge, not much research on this issue has been done.

The first study related to this subject was performed by Cunningham et al. [25] who associated polymorphisms of selected genes (e.g., rs3218203 and rs760607 *E2F2*) with the risk of ovarian cancer and showed this relationship. Next, Justenhoven et al. [12] found that *E2F2* rs760607 promoter polymorphism was not significantly associated with the risk of breast cancer.

Lu et.al [20] analyzed 1096 samples of HNSCC with different locations for three SNPs of *E2F1* (rs3213180, rs3213182, and rs3213183) and seven SNPs of *E2F2* (rs3218121, rs2742976, rs6667575, rs3218203, rs3218148, rs3218211, and rs3218123) and revealed that any of the *E2F1* or *E2F2* variants alone might not have a substantial effect on HNSCC risk, but a joint effect of the combined risk genotypes (i.e., rs3213182 AA, rs3213183 GG, rs3213180 GG, rs3218121 GG, rs2742976 GT+TT, rs6667575 GA+AA, rs3218203 CC, rs3218148 AA, rs3218211 CC, and rs3218123 GT+TT) might contribute to the risk of HNSCC, and this increased risk was more pronounced among the younger adults (≤57 years old), men, never smokers, never drinkers, individuals with a family history of cancer in the first-degree relatives, and patients with oropharyngeal cancer. In our study, we could not investigate the combined effect of polymorphisms on cancer risk due to the small number of samples. Moreover, in the case of rs3218121, a statistically significant disagreement with the Hardy-Weinberg equilibrium could be observed in genotype frequency distribution for both cancer patients and the control group.

Thus, it seems that our study is the first one to show the relationship between each of those five polymorphisms of *E2F2* (i.e., rs3218121, rs3218211, rs3218203, rs6667575, and rs3218148) and the risk of OSCC and OPSCC in the European population. As expected from other literature [9], the most significant factors in our study that could contribute to cancer were gender (females had significantly lower odds of cancer than males, OR = 9.15, *p* < 0.001) and smoking (smoking increased the odds of cancer, OR = 0.11, *p* < 0.001). Our results suggested that the most significant differences between the control group and the cancer group were found in the case of the A/G heterozygote for rs3218121. Samples containing this genotype were mostly observed in the control group.

rs6667575 showed a potentially significant (*p* ≤ 0.1) decrease in the odds of cancer in the case of subjects with the A/G heterozygote. This observation seemed to be additionally supported by the fact that in the whole study cohort, the odds of T = 1 were significantly lower for such subjects, while in subjects with the G/G homozygote the odds were significantly increased. However, such findings were found only in the whole study cohort. Therefore, it is possible that the influence of the G/G homozygote on progression could be of minor importance. In the patient group, however, the presence of A/A homozygote seemed to increase the odds (T = 4).

The A/G heterozygote rs3218121 significantly decreased the odds of cancer (*p* < 0.001). In the case of each TNM staging and grading, the influence of the G/G homozygote could significantly increase the odds of staging and grading, while the A/G heterozygote significantly decreased the odds. However, in our study, there was a significant difference in genotype distribution between the cancer and control groups. Additionally, in the case of cancer patients, only the odds of T = 1 were significantly higher for the A/A homozygote, which could be additionally observed in the whole study cohort. Therefore, rs3218121 should be further analyzed in future studies.

In this study, rs3218211 can be the only polymorphism that showed a consistent increase in the odds of T = 1 for the G/G homozygote in both the cancer and whole cohort groups and a consistent decrease in odds of T = 1 for both the A/G heterozygote and A allele. Surprisingly, however, in the case of the A/A homozygote, we observed an increase in the odds of N = 2 in the whole study cohort, with a corresponding decrease in the odds for the G allele. This may suggest that in the case of progression, the A/G heterozygote could be more beneficial for patients, but the presence of A/A homozygote (and the lack of the G allele) could unfavourably impact cancer progression (node involvement). Despite the above, rs3218211 did not significantly influence the odds of cancer.

The impact of A/G for rs3218148 in cancer patients was limited to a decrease in the odds of G = 2, which could also be found in the combined cohort. However, in the whole study cohort, an additional increase in the odds of G = 2 could be observed for the A/A homozygote, and the presence of the G allele decreased the odds. A similar observation was seen when T = 4 was analyzed. The odds were increased for subjects with the A/A homozygote, while the G allele decreased the odds. This may suggest that in the case of rs3218148 the G allele (and by extension also the heterozygote) could be more beneficial for cancer progression. However, no significant influence of any of rs3218148 genotypes could be seen in the case of cancer.

In our study, rs3218203 had no significant impact on the cancer status.

Our research has limitations that need to be addressed. Firstly, the small sample size has attenuated statistical power. Secondly, since activating E2F2 transcription through its interaction with histone deacetylases (HDACs) can facilitate HPV31 E7 replication [26], it would be reasonable to determine *E2F2* expression and HPV in further studies.

## 5. Conclusions

In conclusion, in our samples, rs6667575, rs3218121, rs3218211, and rs3218148 polymorphisms may affect the course of OSCC and OPSCC, while rs3218203 was not associated with OSCC and OPSCC. However, further studies are warranted to confirm our findings.

## Figures and Tables

**Table 1:** *E2F2* polymorphisms and primers used in the study.

SNP ID	Context sequence [VIC/FAM]
rs6667575	ATAAGACCCTTTTACTCTAGTCTAC[A/G]TATCTCATTGGTCCTTTTTGGTCCT
rs3218121	TCTATTCAGCGCCTACAGGATGCCA[A/G]GCACCATGCTAGATCCTTACAAGCG
rs3218211	GAGGCCTAAGTGCAATTAGCATTCT[A/G]GCAGACTGGACAGCCCCTCAGAGTC
rs3218148	GCTCCTCTCCACCCTGTTGCCACCC[A/G]GGCCCCAATTAGGCCCAGAGCTGCA
rs3218203	GTAGCCTCAGCTTGTCTCCACTTCC[C/G]TATTACTATTCTCTCTTCAACTCAC

**Table 2:** Clinical parameters of patients with OSCC and OPSCC.

Clinical parameters	Patients, *n* (%)
Histological grading	
G1 (well differentiated)	16 (17)
G2 (moderately differentiated)	64 (68)
G3 (poorly differentiated)	14 (15)
T classification	
T1	12 (13)
T2	23 (24)
T3	22 (23)
T4	37 (39)
Nodal status	
N0	42 (45)
N1	24 (25)
N2	26 (28)
N3	2 (2)

**Table 3:** Distribution of *E2F2* polymorphism genotypes in the analyzed patient, controls, and cohort groups.

SNP ID	Genotypes	OSCC and OPSCC+control, *n*	OSCC and OPSCC, *n*	Control, *n*
rs6667575	G/G	84	46	38
	A/G	89	36	53
	A/A	18	10	8
	*p* value	≤1	≤1	≤1
rs3218121	G/G	80	80	0
	A/G	82	3	79
	A/A	31	12	19
	*p* value	≤1	≤0.001	≤0.001
rs3218211	A/A	51	25	26
	G/A	90	40	50
	G/G	51	28	23
	*p* value	≤1	≤1	≤1
rs3218148	G/G	32	15	17
	A/G	105	48	57
	A/A	53	30	23
	*p* value	≤1	≤1	≤1
rs3218203	C/C	128	65	63
	G/C	62	30	32
	G/G	2	0	2
	*p* value	≤0.1	≤1	≤1

**Table 4:** Associations of *E2F2* genotypes with odds of OSCC and OPSCC patients and controls.

SNP ID	Genotypes	OSCC and OPSCC	Controls	*p* value	OR (95% CI)
rs6667575	G/G	46	38	≤1	0.62 (0.35-1.11)
	A/G	36	53	≤0.1	1.79 (1.01-3.19)
	A/A	10	8	≤1	0.72 (0.27-1.91)
	Var. (A)	46	61	≤1	1.61 (0.90-2.85)
	Ref. (G)	82	91	≤1	1.39 (0.52-3.68)
rs3218121	G/G	80	0	—	Absent in control
	A/G	3	79	≤0.001	127.51 (36.38-446.93)
	A/A	12	19	≤1	1.66 (0.76-3.65)
	Var. (A)	15	98	—	G/G homozygote absent in control
	Ref. (G)	83	79	≤1	0.60 (0.27-1.32)
rs3218211	A/A	25	26	≤1	0.97 (0.51-1.84)
	G/A	40	50	≤1	1.35 (0.77-2.39)
	G/G	28	23	≤1	0.70 (0.37-1.34)
	Var. (G)	68	73	≤1	1.03 (0.54-1.96)
	Ref. (A)	65	76	≤1	1.42 (0.75-2.71)
rs3218148	G/G	15	17	≤1	1.11 (0.52-2.37)
	A/G	48	57	≤1	1.34 (0.75-2.37)
	A/A	30	23	≤1	0.65 (0.34-1.24)
	Var. (A)	78	80	≤1	0.90 (0.42-1.94)
	Ref. (G)	63	74	≤1	1.53 (0.81-2.90)
rs3218203	C/C	65	63	≤1	0.86 (0.47-1.56)
	G/C	30	32	≤1	1.07 (0.58-1.95)
	G/G	0	2	—	Absent in cancer
	Var. (G)	30	34	≤1	1.17 (0.64-2.13)
	Ref. (C)	95	95	—	G/G homozygote absent in cancer

## Data Availability

The data used to support the findings of this study are available from the corresponding author upon request.
